# Neuroprotective Effects of the Lithium Salt of a Novel JNK Inhibitor in an Animal Model of Cerebral Ischemia–Reperfusion

**DOI:** 10.3390/biomedicines10092119

**Published:** 2022-08-29

**Authors:** Igor A. Schepetkin, Galina A. Chernysheva, Oleg I. Aliev, Liliya N. Kirpotina, Vera I. Smol’yakova, Anton N. Osipenko, Mark B. Plotnikov, Anastasia R. Kovrizhina, Andrei I. Khlebnikov, Evgenii V. Plotnikov, Mark T. Quinn

**Affiliations:** 1 Department of Microbiology and Cell Biology, Montana State University, Bozeman, MT 59717, USA; 2 Department of Pharmacology, Goldberg Research Institute of Pharmacology and Regenerative Medicine, Tomsk NRMC, 634028 Tomsk, Russia; 3 Department of Pharmacology, Siberian State Medical University, 2 Moskovskiy tract, 634050 Tomsk, Russia; 4 Radiophysical Faculty, National Research Tomsk State University, 634050 Tomsk, Russia; 5 Kizhner Research Center, Tomsk Polytechnic University, 634050 Tomsk, Russia; 6 Research School of Chemistry and Applied Biomedical Sciences, Tomsk Polytechnic University, 634050 Tomsk, Russia

**Keywords:** c-Jun *N*-terminal kinase, 11*H*-indeno[1,2-*b*]quinoxalin-11-one, oxime, interleukin-6, nuclear factor-κB, lithium salt, stroke

## Abstract

The c-Jun *N*-terminal kinases (JNKs) regulate many physiological processes, including inflammatory responses, morphogenesis, cell proliferation, differentiation, survival, and cell death. Therefore, JNKs represent attractive targets for therapeutic intervention. In an effort to develop improved JNK inhibitors, we synthesized the lithium salt of 11*H*-indeno[1,2-*b*]quinoxaline-11-one oxime (**IQ-1L**) and evaluated its affinity for JNK and biological activity in vitro and in vivo. According to density functional theory (DFT) modeling, the Li^+^ ion stabilizes the six-membered ring with the 11*H*-indeno[1,2-*b*]quinoxaline-11-one (**IQ-1**) oximate better than Na^+^. Molecular docking showed that the *Z* isomer of the **IQ-1** oximate should bind JNK1 and JNK3 better than (*E*)-**IQ-1**. Indeed, experimental analysis showed that **IQ-1L** exhibited higher JNK1-3 binding affinity in comparison with **IQ-1S**. **IQ-1L** also was a more effective inhibitor of lipopolysaccharide (LPS)-induced nuclear factor-κB/activating protein 1 (NF-κB/AP-1) transcriptional activity in THP-1Blue monocytes and was a potent inhibitor of proinflammatory cytokine production by MonoMac-6 monocytic cells. In addition, **IQ-1L** inhibited LPS-induced c-Jun phosphorylation in MonoMac-6 cells, directly confirming JNK inhibition. In a rat model of focal cerebral ischemia (FCI), intraperitoneal injections of 12 mg/kg **IQ-1L** led to significant neuroprotective effects, decreasing total neurological deficit scores by 28, 29, and 32% at 4, 24, and 48 h after FCI, respectively, and reducing infarct size by 52% at 48 h after FCI. The therapeutic efficacy of 12 mg/kg **IQ-1L** was comparable to that observed with 25 mg/kg of **IQ-1S,** indicating that complexation with Li^+^ improved efficacy of this compound. We conclude that **IQ-1L** is more effective than **IQ-1S** in treating cerebral ischemia injury and thus represents a promising anti-inflammatory compound.

## 1. Introduction

Cerebral ischemic stroke is caused by cerebral malperfusion due to arterial insufficiency. This can result in acidotoxicity, excitotoxicity, oxidative stress, and inflammation, with subsequent alteration of various cellular functions [1,2,3]. Ischemic stroke is the most common type of stroke and can lead to an acute and progressive neurodegenerative condition with limited functional recovery and high mortality [4].

The inflammatory response to acute cerebral ischemia is one of the main pathophysiological factors that determine the outcome of ischemic stroke [5]. Inflammation caused by impaired blood supply to the brain is accompanied by the activation of intravascular leukocytes and the release of proinflammatory mediators from the ischemic endothelium and brain parenchyma, which can increase tissue damage [5,6]. Immune cells, including T cells, B cells, dendritic cells, neutrophils, macrophages, and microglial cells, are important in the regulation and progression of ischemic brain injury [6,7,8]. Cerebral ischemia induces inflammatory responses, such as neutrophil infiltration, cytokine production, and microglial activation [9,10]. Several studies have demonstrated that administration of immunomodulators and anti-inflammatory compounds acting in the brain may represent new therapeutic strategies to treat acute ischemic stroke [11,12,13,14].

The c-Jun *N*-terminal kinases (JNKs) are important mitogen-activated protein kinases activated by various brain insults and are involved in neuronal injury triggered by reperfusion-induced oxidative stress [2,15,16,17]. Three distinct JNKs, designated as JNK1, JNK2, and JNK3, have been identified, and at least 10 different splicing isoforms exist in mammalian cells [18]. JNK3 is found almost exclusively in the brain, but it is not dominant, as JNK3 knockout results only in weak attenuation of the total JNK pool in brain tissue [19,20]. In any case, the JNKs represent important targets for the development of therapeutic treatments because these kinases play important roles in regulating inflammation, the signaling events leading to apoptosis, necrosis, and transcriptional and non-transcriptional processes involved in the injury of neurons during ischemia and reperfusion [2,21,22]. For example, increased JNK phosphorylation and JNK activity in the hippocampus have been reported in the brains of rats and mice after global and focal cerebral ischemia (FCI) [23,24,25,26]. Sustained JNK activation has also been shown to be associated with neuronal death and apoptosis following ischemic stroke and acute inhibition of JNK reduced infarction and improved outcomes in animal models of cerebral ischemia [15,16]. Because the inhibition of JNK has neuroprotective effects in animal models, it has been suggested that JNK inhibitors may represent promising therapeutic agents for the treatment of stroke [22]. To date, various small-molecule synthetic inhibitors of JNK enzymatic activity have been described [17,25,27,28,29], and some of these compounds have demonstrated neuroprotective activity in the animal models of stroke [15,30,31,32].

The specific JNK inhibitor, 11*H*-indeno[1,2-*b*]quinoxalin-11-one oxime (**IQ-1**), and several analogs of this compound have been shown to exhibit high affinity for JNK3 compared with their affinities for JNK1/JNK2 [27,33]. **IQ-1** and its Na^+^ salt (i.e., **IQ-1S**) have been shown to exhibit immunomodulatory and anti-inflammatory effects in cell culture and animal models of inflammation [27,33,34], as well as antiviral activity against SARS-CoV-2 [35]. Recently, we found that **IQ-1S** exhibited neuroprotective activity in models of FCI in mice and rats [36], as well as in a model of global cerebral ischemia in rats [37].

Since oximes, including derivatives of 11*H*-indeno[1,2-*b*]quinoxalin-11-ones and related tryptanthrin analogs, could form complexes with different metal ions, and the ion-chelating properties of ligands can alter to their biological activities [38,39,40], we hypothesized that other salts of **IQ-1** may lead to a change in therapeutic potential of the resulting ion complexes. In particular, we evaluated lithium salts of **IQ-1** because Li^+^ is also a promising candidate for the treatment of ischemic stroke [41,42,43,44,45,46,47,48,49]. Moreover, Li^+^ is used as a first-line medication in the treatment of bipolar disorder [50], and Li^+^ ameliorates inflammation and stimulates neuroregeneration in experimental models of Parkinson’s disease, traumatic brain injury, and Alzheimer’s disease (for review see [51,52]). Thus, we conducted molecular modeling of Li^+^ and Na^+^ salts of **IQ-1** (**IQ-1L** and **IQ-1S**, respectively) and revealed some differences in the structure of ion-chelating conformations of **IQ-1**. We also found that **IQ-1L** had ~2-fold higher binding activity affinity for JNK1-3 in comparison with **IQ-1S**. Importantly, **IQ-1L** was also generally more effective than **IQ-1S** in inhibiting monocyte/macrophage nuclear factory κB (NF-κB) activity. Finally, **IQ-1L** demonstrated neuroprotective activity in a model of reversible FCI in rats with higher therapeutic efficacy compared to that of **IQ-1S**.

## 2. Materials and Methods

### 2.1. Animals

This research was performed in accordance with the EU Directive 2010/63/EU concerning the protection of animals used for scientific purposes and was approved by the Animal Care and Use Committee of the Goldberg Research Institute of Pharmacology and Regenerative Medicine, Tomsk NRMC (protocol No 130092017 from 08.09.17). Experiments were performed on 29 adult male Wistar rats (weight: 250–280 g). Rats were housed in groups of five animals per cage (57 × 36 × 20 cm) under standard laboratory conditions (ambient temperature of 22 ± 2 °C, relative humidity of 60%, and 12:12 h light–dark cycle) in cages with sawdust bedding and provided with standard rodent feed and *ad libitum* water access.

### 2.2. Compound Synthesis

**IQ-1L** was prepared from **IQ-1** (0.247 g, 1.0 mmol) by treatment of **IQ-1** with an excess of LiOH (0.036 g, 1.5 mmol) in refluxing EtOH or MeOH (10 mL) for 3 h with thin-layer chromatography monitoring. After cooling, the precipitate was filtered and recrystallized from EtOH to yield the lithium salt of oxime 11*H*-indeno[1,2-*b*]quinoxaline-11-one (**IQ-1L**). Yield 33%, colorless crystals. M.p. 399 °C. ^1^H NMR (400 MHz, DMSO-d_6_), d, ppm: 7.71–7.73 (m, 2H, H-2, H-3), 7.83–7.86 (m, 2H, H-7, H-8), 8.15 (d, 1H, ^3^J = 1.5 Hz, H-9), 8.17 (d, 1H, ^3^J = 1 Hz, H-6), 8.21 (dd, 1H, ^4^J = 1.5 Hz, ^3^J = 4 Hz, H-4), 8.58 (d, 1H, ^3^J = 2.4 Hz, H-1). ^13^C NMR (100 MHz, DMSO-d_6_), δ, ppm: 122.49; 129.03; 129.63; 130.17; 130.75; 132.29; 132.76; 133.39; 136.34; 141.87; 142.18; 147.45; 151.11; 153.2. Found, %: C 70.93, H 3.18, N 16.37. C_15_H_8_LiN_3_O. Calculated, %: C 71.16, H 3.18, N 16.60. Purity of the sample was 99.9%.

**IQ-1S** was synthesized as described previously [27].

### 2.3. Cerebral Ischemia Model

The model of FCI/reperfusion was induced in rats by intraluminal occlusion of the left middle cerebral artery as described previously [53], with minor modifications. To manage general anesthesia with Propofol-Lipuro (B. Braun Medical Inc., Melsungen, Germany) (10 mg/kg/h), animals were implanted with a catheter placed in the right femoral vein under brief anesthesia with ethyl ether. FCI was achieved by using filaments manufactured by Doccol Corporation (Sharon, MA, USA). Briefly, the left common carotid artery (CCA) was exposed, and the internal carotid artery (ICA) was isolated up to the origin of the pterygopalatinum artery. Likewise, the external carotid artery (ECA) was isolated up to the origin of the lingual and external maxillar arteries. The ICA and ECA were tied with one ligature, and after another ligation proximal to the first, the ECA was crossed between the ligatures, after which microvascular clips were placed across both the CCA and ICA. The ECA stump was immobilized, and the filament was introduced through a small incision and fixed by slightly tightening a ligature. The clips were then removed from the CCA and ICA. After a 1 h occlusion, the filament was extracted, and the ECA was ligated. The blood supply was resumed through the ICA. The group of sham-operated animals received a similar surgical procedure but without filament introduction. The body temperature of rats was maintained at 37.0 ± 0.05 °C during the surgery by a temperature control unit and homeothermic blanket control (Harvard Apparatus, Holliston, MA, USA). After wound closure and anesthesia recovery, animals were returned to their cages with free access to food and water.

### 2.4. Ischemia/Reperfusion Protocol

The group of sham-operated animals was comprised of five rats. The animals with FCI were assigned to two groups. Rats in the control group (*n* = 13) received 2 mL of physiological saline solution containing 20 µL of Tween 80 intraperitoneally (*i.p*.) on 30 min of ischemia as well as 24 h and 48 h after the FCI procedure. Rats in the experimental groups (*n* = 11) received a 2 mL suspension of physiological saline solution containing 20 µL of Tween 80 and **IQ-1L** at doses of 12 mg/kg using the same regimen. Neurological status was assessed in rats at 4, 24, and 48 h after inducing FCI. The size of cerebral infarction was assessed 48 h after reperfusion. The rats were euthanized by CO_2_ euthanasia device.

### 2.5. Neurological Deficit Evaluation

Experimenters unaware of the group the animals were assigned to examine neurological status of the animals. The degree of neurological deficit in the FCI model was determined by the method of Zhang et al. [54], which is based on the tail suspension test (graded from 0–3), posture maintenance (graded from 0–2), circling test (graded from 0–3), and horizontal righting reflex (graded from 0–3). The neurological deficit was calculated as the total score from all tests, which ranged from 0–11, with 0 representing normal behavior and 11 indicating severely impaired neurological function.

### 2.6. Assessment of Cerebral Infarct Size

To measure infarct size after FCI, brains were frozen at –12 °C for 2.5 h, and 1.3 mm-thick frontal brain slices were prepared using a series of histological knives. After being thawed to room temperature, the brain slices were incubated in a solution of 0.5% 2,3,5-triphenyltetrazolium chloride (TTC) (Sigma-Aldrich Chemical Co., St. Louis, MO, USA) at 37 °C in the dark for 15 min. The brain slices were then fixed in 10% buffered formalin for 15 min, placed on glass slides, and scanned at 600 dpi resolution using a HP Scanjet 3770 (Hewlett-Packard, China) with HP Director software v. 43.1.6.000. Images were stored in *.tiff format and processed with Adobe Photoshop 6.0 software. The cerebral infarction area and total area of the brain slice were calculated for each slide. The cerebral infarction area is expressed as a percentage of the total area of the slices.

### 2.7. Kinase *K*_d_ Determination

**IQ-1L** was submitted for dissociation constant (*K*_d_) determination using KINOMEscan (Eurofins Pharma Discovery, San Diego, CA, USA), as described previously [55]. In brief, kinases were produced and displayed on T7 phage or expressed in HEK-293 cells. Binding reactions were performed at room temperature for 1 h, and the fraction of kinase not bound to the test compound was determined by capture with an immobilized affinity ligand and quantified by quantitative polymerase chain reaction. Primary screening at fixed concentrations of compound was performed in duplicate. For dissociation constant *K*_d_ determination, a 12-point half-log dilution series (a maximum concentration of 33 μM) was used. Assays were performed in duplicate, and their average mean value is displayed.

### 2.8. Cell Culture

All cells were cultured at 37 °C in a humidified atmosphere containing 5% CO_2_. THP-1Blue cells obtained from InvivoGen (San Diego, CA, USA) were cultured in RPMI 1640 medium (Mediatech Inc., Herndon, VA, USA) supplemented with 10% (*v*/*v*) fetal bovine serum (FBS), 100 μg/mL streptomycin, 100 U/mL penicillin, 100 μg/mL phleomycin (Zeocin), and 10 μg/mL blasticidin S. Human monocyte–macrophage MonoMac-6 cells (Deutsche Sammlung von Mikroorganismen und Zellkulturen GmbH, Braunschweig, Germany) were grown in RPMI 1640 medium supplemented with 10% (*v*/*v*) FBS, 10 μg/mL bovine insulin, 100 μg/mL streptomycin, and 100 U/mL penicillin.

### 2.9. Analysis of AP-1/NF-κB Activation

Activation of AP-1/NF-κB was measured using an alkaline phosphatase reporter gene assay in THP1-Blue cells. Human monocytic THP-1Blue cells were stably transfected with a secreted embryonic alkaline phosphatase gene that was under the control of a promoter inducible by NF-κB/AP-1. THP-1Blue cells (2 × 10^5^ cells/well) were pretreated with test compound or dimethyl sulfoxide (DMSO; 1% final concentration) for 30 min, followed by addition of 250 ng/mL lipopolysaccharide (LPS; from *Escherichia coli* strain 0111:B4) for 24 h, and alkaline phosphatase activity was measured in cell supernatants using QUANTI-Blue mix (InvivoGen) as absorbance at 655 nm and compared with positive control samples (LPS). The concentration of compound that caused 50% inhibition of the NF-κB reporter activity (IC_50_) was calculated.

### 2.10. Cytokine Analysis

A human IL-6 ELISA kit (BD Biosciences, San Jose, CA, USA) was used to confirm the inhibitory effect of **IQ-1L** on IL-6 production. MonoMac-6 cells were plated in 96-well plates at a density of 2 × 10^5^ cells/well in culture medium supplemented with 3% (*v*/*v*) endotoxin-free FBS. Cells were pretreated with the compound or 1% DMSO (negative control) for 30 min, followed by addition of 250 ng/mL LPS for 24 h. IC_50_ for IL-6 production was calculated by plotting percentage inhibition against the logarithm of inhibitor concentration (at least five points). A multiplex human cytokine ELISA kit from Anogen (Mississauga, ON, Canada) was also used to evaluate interleukin (IL)-1α, IL-1β, IL-6, tumor necrosis factor (TNF), monocyte chemoattractant protein-1 (MCP-1), interferon-γ (IFN-γ), and granulocyte–macrophage colony-stimulating factor (GM-CSF) in MonoMac-6 cell supernatants.

### 2.11. Cytotoxicity Assay

Cytotoxicity was analyzed with a CellTiter-Glo Luminescent Cell Viability Assay Kit from Promega (Madison, WI, USA), according to the manufacturer’s protocol. Cells were treated with varying concentrations of **IQ-1L** (up to 50 µM) and cultivated for 24 h. After treatment, the cells were allowed to equilibrate to room temperature for 30 min, substrate was added, and the samples were analyzed with a Fluoroscan Ascent FL (Thermo Fisher Scientific, Waltham, MA, USA). The cell IC_50_ was calculated by plotting percentage inhibition against the logarithm of compound concentration (at least five points).

### 2.12. Western Blotting

MonoMac-6 monocytic cells (10^7^ cells) were incubated with different concentrations of **IQ-1L** (final DMSO 0.5%) for 30 min at 37 °C and then treated with LPS (250 ng/mL) or buffer for another 30 min at 37 °C. Cells were washed twice with ice-cold phosphate buffer solution (pH 7.4), and cell lysates were prepared using lysis buffer (Cell Signaling Technology, Danvers, MA, USA). Cell lysates were separated on ExpressPlus 10% PAGE Gels (GenScript, Piscataway, NJ, USA) using TRIS-MOPS running buffer and transferred to nitrocellulose membranes. The blots were blocked overnight at 4 °C in TRIS buffer (pH 7.4) + 0.1% Tween-20 (TBST) + 2.5% bovine serum albumin and probed with antibodies against phospho-c-Jun (Ser63) (Cell Signaling Technology), followed by horseradish peroxidase-conjugated secondary antibody (Cell Signaling Technology), and the blots were developed using Super-Signal West Femto chemiluminescent substrate (Thermo Fisher Scientific) and visualized with a FluorChem FC2 imaging system (Alpha Innotech Corporation, San Leandro, CA, USA). For measurement of total c-Jun signal, we reprobed the same Western blots that were used for phospho-c-Jun blots. Briefly, the membranes were washed 4 times for 5 min with TBST, incubated for 30 min at 50 °C in TRIS buffer (pH 6.3) + 2% sodium dodecyl sulfate + 0.63% β-mercaptoethanol, and then were washed 6 times for 5 min each in TBST. The membranes were blocked again and probed for total c-Jun, followed by horseradish peroxidase-conjugated secondary antibody (both reagents from Cell Signaling Technology), developed, and visualized as described above. Quantitation of the chemiluminescent signals were performed using AlphaView software.

### 2.13. Molecular Modeling

DFT calculations were performed using ORCA quantum chemistry software [56], version 5.0.2. The composite method r^2^SCAN-3c [57] was applied for geometry optimization. The CPCM solvation model [58] was used with water as a solvent. All the stationary points on the potential energy surface after the optimization were classified as minima based on frequency analysis.

Geometry of JNK3 was obtained by downloading the crystal structure from the Protein Data Bank (PDB entry code 1PMV) into Molegro software (Molegro ApS, Aarhus, Denmark). All solvent molecules were removed. A search space was chosen for the receptor as a sphere centered on co-crystallized ligand present in the corresponding PDB structure. The radius of the sphere was equal to 10 Å. The sphere completely encompassed the co-crystallized ligand and the binding site. Side chains of all amino acid residues of a receptor within the corresponding sphere were regarded as flexible during docking. The number of such residues was equal to 39. The flexible residues were treated with default settings of the “Setup Sidechain Flexibility” tool in Molegro, and a softening parameter of 0.7 was applied during flexible docking according to the standard protocol using the Molegro Virtual Docker 6.0 (MVD) program. Before docking, the structure of the **IQ-1** *E* isomer was pre-optimized using HyperChem software (HyperCube, Gainesville, FL, USA) with the MM+ force field and saved in Tripos MOL2 format (Tripos, St. Louis, MO, USA). The ligand structure was imported into MVD. The options “Create explicit hydrogens”, “Assign charges (calculated by MVD)”, and “Detect flexible torsions in ligands” were enabled during importing. Appropriate protonation states of the ligands were also automatically generated at this step. The ligand was subjected to 30 docking runs with respect to a given receptor structure using MVD software. The docking poses obtained were saved together with the corresponding optimal geometries for identified flexible residues.

### 2.14. Statistical Analysis

Statistical analysis was performed with Statistica 8.0 software. Results of in vivo experiments are expressed as mean ± SEM. Group variation was assessed with a Kruskal–Wallis test. Significant differences between the variables were assessed by Mann–Whitney U-test. Compliance of the sample with normal distribution was evaluated by Shapiro–Wilk’s W test and Kolmogorov–Smirnov and Lilliefors test. Results of all in vitro experiments are expressed as mean ± SD. Values were considered statistically significant when *p* was < 0.05.

## 3. Results and Discussion

### 3.1. Molecular Modeling

**Figure 1:**
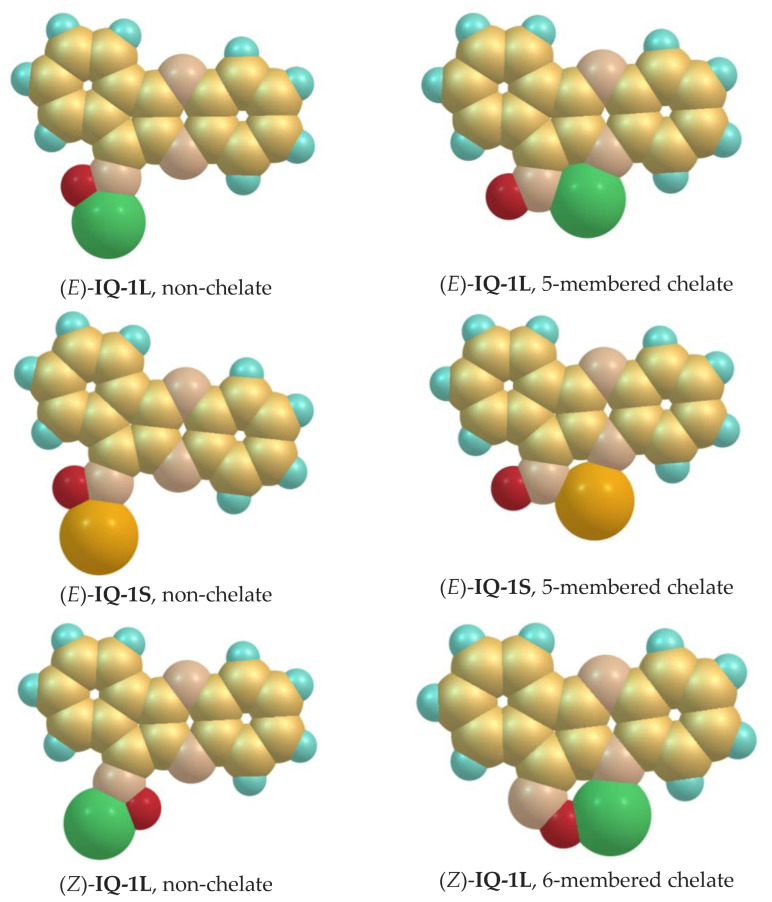

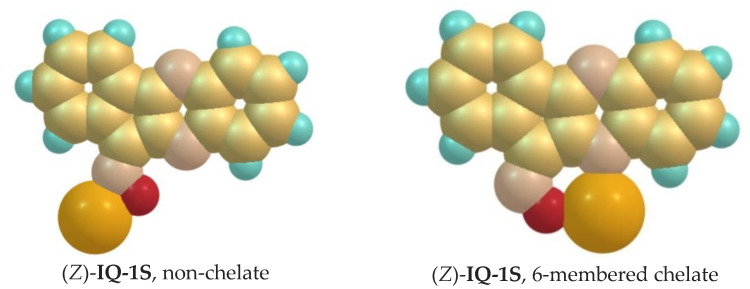
Low-energy geometric structures of the Na^+^ and Li^+^ salts of **IQ-1** in non-chelated and chelated forms of their *E* and *Z* isomers.

**Scheme 1:**
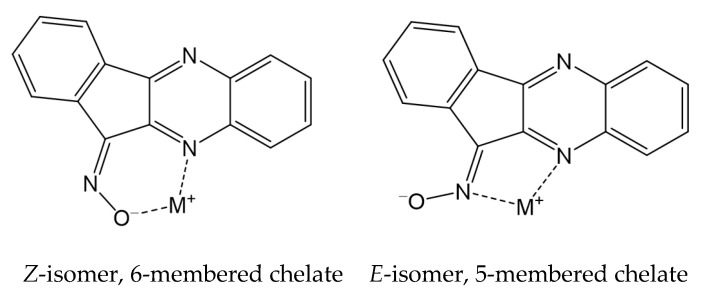
Structural formulae of **IQ-1L** and **IQ-1S** lower-energy chelated forms (M = Li, Na).

Although the Na^+^ and Li^+^ salts of **IQ-1** are very similar compounds from a chemical point of view, there should be differences in their structures owing to significantly different cation radii. To evaluate the interaction between each of the cations and the **IQ-1** oximate ion, we performed density functional theory (DFT) calculations on **IQ-1L** and **IQ-1S**. The optimized geometries and thermodynamic quantities were calculated by the composite DFT method r^2^SCAN-3c [57] with the CPCM solvation model. Analysis of these structures demonstrated that both *Z* and *E* isomers of the salts were more stable in the form in which the metal cation was chelated by the oxygen atom of the oximate and the nitrogen atom of the heterocycle, or by two nitrogen atoms, one each from the oximate and the heterocycle, respectively (Table 1). Stabilization by chelation is especially effective in the case of *Z* isomers, which is obviously due to participation of the oxygen atom and the formation of 6-membered chelates (as opposed to 5-membered chelates for the *E* isomers) (Figure 1, Scheme 1). Moreover, Li^+^ stabilizes the six-membered ring in the **IQ-1** oximate better than the Na^+^. Note that the *Z* isomer also has much lower Gibbs free energy than the corresponding *E* isomer, and more positive values of ΔG were observed for the transition of the *Z* isomer to the *E* isomer for the Li^+^ salt.

The results suggest that chelation may play an important role in the relative isomer stabilities. As oximates are susceptible to a rapid hydrolysis into the corresponding oximes in aqueous medium, **IQ-1** is presumably the actual molecule that interacts to JNK. Thus, hydrolysis of **IQ-1L** should lead to a higher *Z*/*E* ratio of **IQ-1** as compared with the hydrolysis of **IQ-1S**. Indeed, the *Z*,*E* isomerization of oximes is characterized by high energy barriers and is a relatively slow process [59].

**Figure 2:**
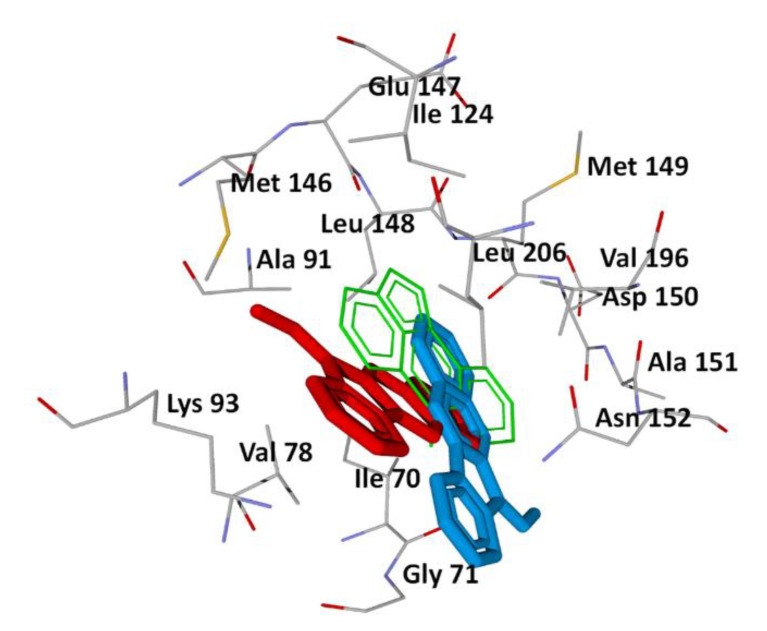
Docking poses of (*Z*)-**IQ-1** (blue) and (*E*)-**IQ-1** (red) into the JNK3 binding site (PDB: 1PMV). The co-crystallized ligand SP600125 is shown in thin green lines. Residues within 5 Å from SP600125 are visible.

In order to evaluate the ability of **IQ-1** to bind JNK3 in the form of either the *Z* or *E* isomer, we performed molecular docking of these isomers into the enzyme binding site. It should be noted that in our previous study [27], we docked (*Z*)-**IQ-1** using the structure of JNK3 co-crystallized with JNK inhibitor SP600125 [60] and obtained a docking pose in which the molecule formed H-bonds with Ile70 and Asn152 of JNK3. Here, we report docking results of (*E*)-**IQ-1** obtained with the same enzyme structure and MVD software options as we used for the *Z* isomer. We found that (*E*)-**IQ-1** occupies the narrow cleft of JNK3 near the co-crystallized SP600125 molecule; however, it did not form H-bonds with the enzyme and was oriented differently within the ligand binding site (Figure 2) compared with (*Z*)-**IQ-1**. In addition, the calculated MolDock docking score (D_s_) for the *E*-isomer was less favorable for binding to JNK3: D_s_((*E*)-**IQ-1**) = −52.56, D_s_((*Z*)-**IQ-1**) = −68.52 (expressed in MolDock force field units [61]). Thus, according to the docking results, the *Z* isomer, which should be abundant in the case of the more active Li^+^ salt, has better binding affinity for JNK3 than (*E*)-**IQ-1**.

Although JNK3 is relatively specifically expressed in brain [62], JNK1 is also an important molecular target for stroke treatment [63,64]. Previously, we also conducted a molecular docking of **IQ-1** into structure of JNK1 and found that docking of the *Z* isomer gave the best pose, which was almost identical to that of the co-crystallized JNK inhibitor SP600125 and in contrast to that of the *E* isomer [33].

### 3.2. Affinity of IQ-1L for JNK

**IQ-1L** was evaluated for its ability to bind to JNK1-3, and these data were compared with the previously reported JNK affinity of **IQ-1S** [27]. We used the KINOMEscan ATP site-dependent binding assay, which reflects the biologically relevant behavior of protein kinases [55,65]. We found that **IQ-1L** bound to JNK1, JNK2, and JNK3 with *K*_d_ values of 0.14 ± 0.01, 0.15 ± 0.04, and 0.055 ± 0.01 μM, respectively. These values are ~2-fold lower than those reported for **IQ-1S** (0.39, 0.36, and 0.09 μM for JNK1-3, respectively) [27], indicating that **IQ-1L** has a higher binding affinity for JNK in comparison with its Na^+^ salt, which is consistent with the molecular docking results reported above (see Figure 2).

**Figure 3:**
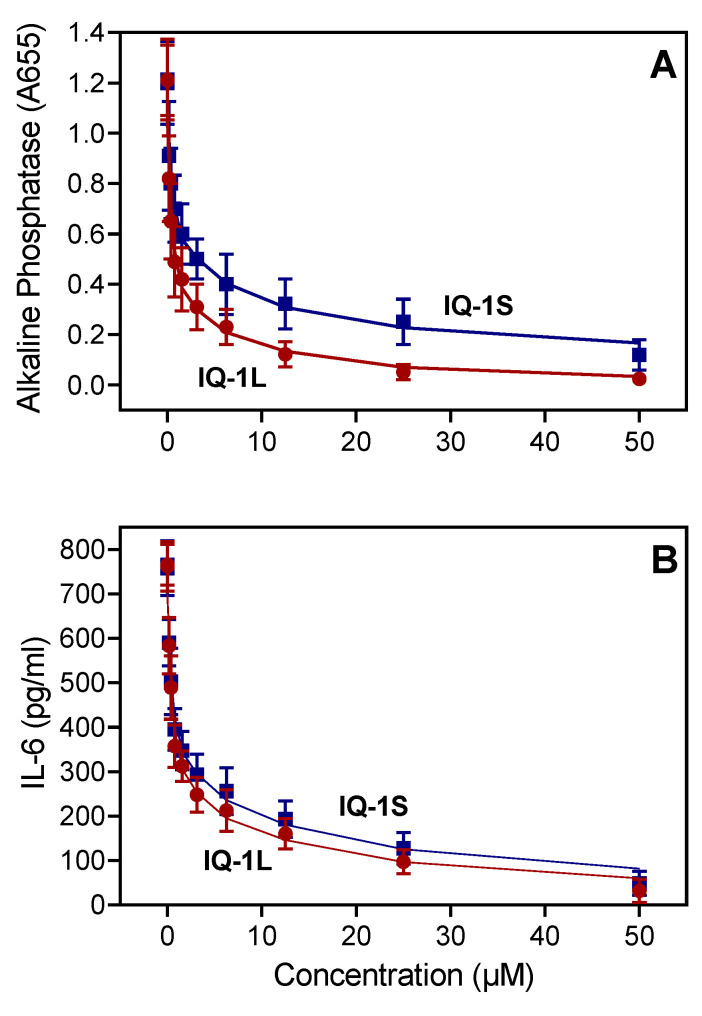
Effect of **IQ-1L** and **IQ-1S** on NF-κB/AP-1 activity and IL-6 production. (**A**) THP-1Blue cells were pretreated with the indicated compounds or DMSO for 30 min, followed by addition of 250 ng/mL LPS or buffer for 24 h. NF-kB/AP-1 activity was monitored by measuring secreted alkaline phosphatase activity spectrophotometrically in the cell supernatants (absorbance at 655 nm). (**B**) MonoMac-6 cells were pretreated with the indicated compounds or DMSO for 30 min, followed by addition of 250 ng/mL LPS or buffer for 24 h. Production of IL-6 in the supernatants was evaluated by ELISA. The data in each panel are presented as the mean ± S.D. of triplicate samples from one experiment that is representative of three independent experiments.

The JNK pathway can be activated through the Toll-like receptor 4 (TLR4), leading to the activation of transcription factors NF-κB and AP-1 (reviewed in [66,67,68]). To assess the anti-inflammatory activity of **IQ-1L**, we evaluated its ability to inhibit LPS-induced NF-κB/AP-1 activation in human THP-1Blue monocytes. We found that **IQ-1L** dose-dependently inhibited NF-κB/AP-1 activity with an IC_50_ of 0.5 ± 0.1 μM (Figure 3A), which was significantly more effective than **IQ-1S** (IC_50_ = 1.8 ± 0.3 μM) [27] (*p* < 0.05). Likewise, **IQ-1L** effectively inhibited IL-6 production by human MonoMac-6 monocyte/macrophages cells (Figure 3B), although there was not a significant difference between the effectiveness of **IQ-1L** and **IQ-1S** in this assay (IC_50_ = 0.7 ± 0.2 versus 0.61 ± 0.15 μM, respectively). Note that Li^+^ itself (added as LiCl) had no effect on cell viability, LPS-induced NF-κB/AP-1 transcriptional activity in THP1-Blue cells, or LPS-induced production of IL-6 in MonoMac-6 cells at concentrations up to 50 μM (data not shown).

**Figure 4:**
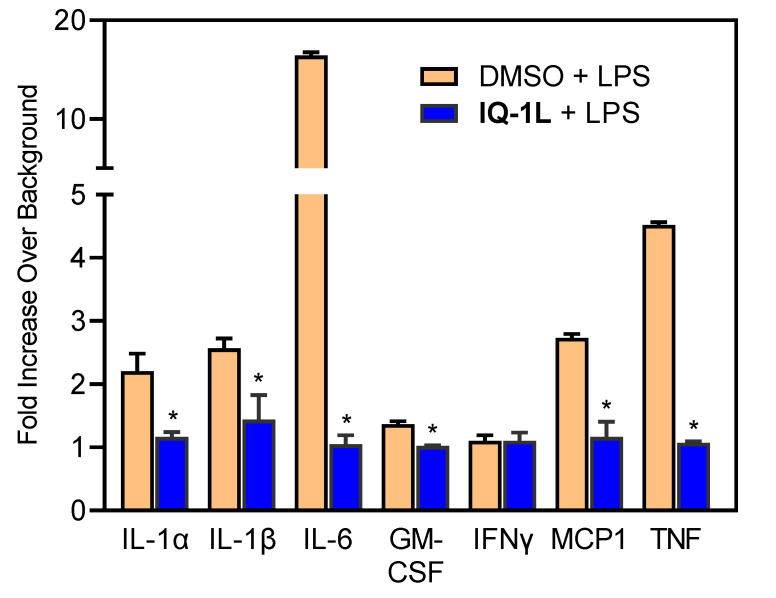
Effect of the **IQ-1L** on LPS-induced proinflammatory cytokine production in MonoMac-6 cells. Human MonoMac-6 monocytic cells were pretreated with 10 μM of **IQ-1L** or 0.5% DMSO for 30 min, followed by addition of 250 ng/mL LPS (**IQ-1L** + LPS) or control buffer for 24 h. Production of cytokines in the supernatants was evaluated using a Multiplex human cytokine ELISA kit. The relative level of cytokine production is shown as fold increase over background (DMSO control). * *p* < 0.01 compared with DMSO + LPS-treated control cells. The data are presented as the mean ± S.D. of triplicate samples from one experiment that is representative of two independent experiments.

Based on the ability of **IQ-1L** to inhibit LPS-induced IL-6 in MonoMac-6 cells, we further analyzed the effect of **IQ-1L** on LPS-induced production of a range of proinflammatory cytokines by MonoMac-6 cells, including IL-6, using a cytokine array. As shown in Figure 4, treatment of MoncMac-6 cells with 250 ng/mL LPS induced IL-1α, IL-1β, IL-6, granulocyte–macrophage colony-stimulating factor (GM-CSF), monocyte chemoattractant protein-1 (MCP1), and TNF in the cells compared with DMSO-treated control cells. Notably, the production of these cytokines was significantly inhibited by treatment with 10 μM **IQ-1L** (Figure 4).

**Figure 5:**
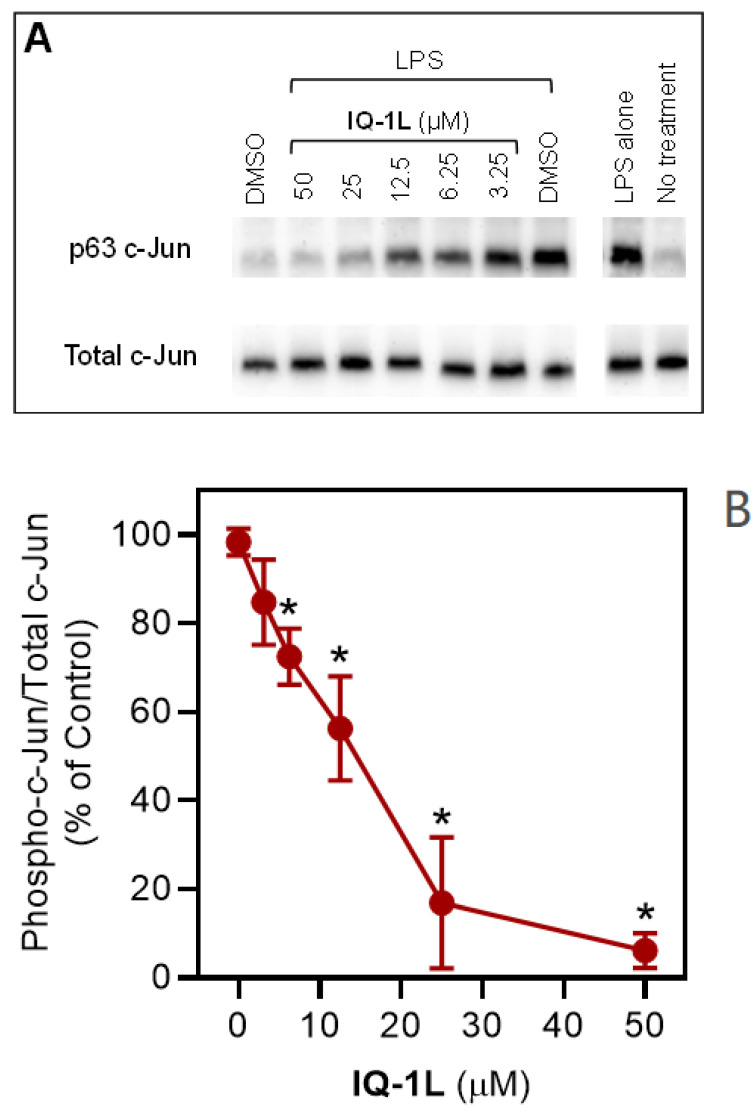
Effect of **IQ-1L** on LPS-induced c-Jun (Ser63) phosphorylation. Human MonoMac-6 monocytic cells were pretreated with indicated concentrations of **IQ-1L** or 0.5% DMSO for 30 min, followed by treatment with LPS (250 ng/mL) or control buffer for another 30 min. Controls with cells alone (no DMSO) or cells treated with LPS alone were also included. The cells were lysed, and the lysates were analyzed by Western blotting on 10% SDS-PAGE gels. After blotting for phospho-c-Jun, the same blots were stripped and reblotted for total JNK as a loading control for the lysates. Representative blots from three independent experiments are shown (**A**). The blots were analyzed by densitometry, as described under *Materials and Methods*, and the ratio of phospho-c-Jun/total c-Jun is shown in (**B**). Values are expressed as mean ± S.D. of three independent experiments. * *p* < 0.05 compared with DMSO + LPS-treated control cells.

To confirm that **IQ-1L** inhibits JNK in cells, we evaluated its effect on c-Jun phosphorylation in MonoMac-6 cells. These cells were pretreated with **IQ-1L**, stimulated with LPS, and the level of phospho-c-Jun (Ser63) was determined. As expected, **IQ-1L** inhibited c-Jun phosphorylation in LPS-treated cells (Figure 5).

Prior to the evaluation of the biological activity of **IQ-1L** in cellular assays, we evaluated the cytotoxicity of **IQ-1L** in human monocytic THP-1Blue and MonoMac-6 cells following a 24 h incubation. **IQ-1L** had no effect on cell viability at concentrations up to 50 μM (data not shown), which is similar to the lack of cytotoxicity reported previously for **IQ-1** and **IQ-1S** [27].

### 3.3. Therapeutic Effects of IQ-1L in FCI

We evaluated the therapeutic efficacy of **IQ-1L** in a model of FCI. In sham-operated animals (*n* = 5), surgical intervention did not lead to changes in neurological status at 4, 24, and 48 h following the procedure (score of neurological deficit = 0). In the control saline-treated group, 3 out of 15 rats died within 48 h during the first day (4–24 h), whereas 1 out of 11 rats died on the second day after FCI in the experimental **IQ-1L**-treated group.

**Figure 6:**
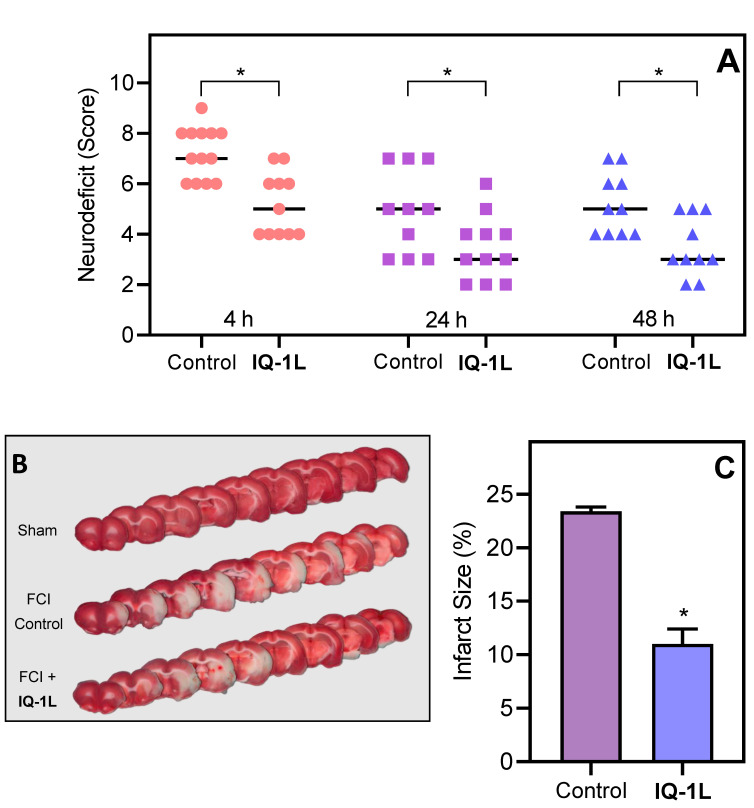
Effects of **IQ-1L** on neurological deficit 4, 24, and 48 h after FCI (**A**) and infarct size in rat brains 48 h after FCI (**B**,**C**). White areas represent the infarct regions in sections of representative samples of stained brain sections from rats sacrificed 48 h after FCI (**B**). * *p* < 0.05: as compared with control animals (FCI without **IQ-1L** treatment).

In the control group of rats, neurological deficiency developed with a severity of 7.2 ± 0.3 at 4 h after FCI. After 24 h and 48 h, there was a spontaneous decrease in the severity of neurological deficit, and the average score of neurological deficit was 4.9 ± 0.6 and 5.2 ± 0.8, respectively. In animals of the experimental **IQ-1L**-treated group (12 mg/kg, *i.p.*), there was a statistically significant decrease in the degree of neurological deficit at all time periods of the study compared with the control group. At 4 h, 24 h, and 48 h after FCI, the mean neurological deficits score were 5.2 ± 0.4, 3.5 ± 0.4, and 3.5 ± 0.4, respectively, which were 28%, 29%, and 33% lower than in the saline-treated control group (Figure 6A).

Macrofocal cerebral infarction formed in all animals of the control group 48 h after FCI. The infarct size was 23.4 ± 0.4% of the total area of the brain sections. In rats of the **IQ-1L**-treated group, infarct size was 11.0 ± 1.4% of the total area of brain sections, which was significantly lower than the corresponding size of the control group (Figure 6B,C). Interestingly, the therapeutic effect of 12 mg/kg **IQ-1L** was comparable to the effect observed for 25 mg/kg of **IQ-1S** [69], indicating that **IQ-1L** had greater efficacy for treatment of FCI. Whether this increased therapeutic efficacy is due to the higher affinity of **IQ-1L** for JNK or its greater ability to inhibit monocyte/macrophage inflammatory responses is not yet clear. Interestingly, we did not observe a difference between **IQ-1S** and **IQ-1L** in their effect on IL-6 production (Figure 3B). IL-6 has been reported to play a role in the pathophysiology of delayed cerebral ischemia [70] but may not be as important in acute FCI. In addition, the hydrolysis of **IQ-1L** releases Li^+^ ions, although the dose (0.047 mEq/kg calculated as the amount of Li^+^ in treated animal) is relatively low in comparison with previously reported doses that exhibit neuroprotective effects in animal models of brain ischemia (1–3 mEq/kg) [44,45,46,47,48]. However, we also cannot exclude possible synergistic effects between **IQ-1** and Li^+^. Li^+^ has a complex (including synergistic) action on different pathways that are associated with ischemia–reperfusion injury. For example, Li^+^ enhanced the anti-ischemic effects of prostaglandins A1 and E1 in rats in a permanent middle cerebral artery occlusion model [71,72,73]. The mechanisms underlying Li^+^-induced neuroprotection may include inactivation of N-methyl-D-aspartate (NMDA) receptors [74,75], reduction in apoptotic cell death by decreasing the level of proapoptotic p53 and enhancing anti-apoptotic Bcl-2 and HSP70 [44,47], activation of the phosphoinositide 3-kinase (PI3K)/Akt cell survival pathway [76], and inhibition of hypoxia-induced glycogen synthase kinase-3 (GSK-3) activation [77].

To conclude, we synthesized and characterized the Li^+^ salt of **IQ-1**. Although previous studies on **IQ-1** utilized the Na^+^ salt (**IQ-1S**), the current molecular modeling studies now suggest that the Li^+^ ion stabilizes the six-membered ring in the **IQ-1** oximate better than Na^+^ ion and that this leads preferably to the presence of the *Z* isomer, which has a higher affinity for JNK1-3 in comparison with **IQ-1S**. **IQ-1L** was a more effective inhibitor of LPS-induced nuclear NF-κB/AP-1 activation in human monocytic cells and was a potent inhibitor of proinflammatory cytokine production by human MonoMac-6 monocyte/macrophages. Moreover, the therapeutic efficacy of **IQ-1L** in the stroke FCI model was higher in comparison with **IQ-1S**, suggesting that the Li^+^ complex represents an improved formulation over the original **IQ-1S** formulation. Additional studies are necessary to define the beneficial therapeutic properties of **IQ-1L** in other experimental models of neuroinflammation.

## Figures and Tables

**Table 1:** Relative Gibbs energies of **IQ-1L** and **IQ-1S** *E* and *Z* isomers calculated by the DFT method.

Cation (M^+^)	* Z * Isomer, Chelate vs. Non-Chelate	* E * Isomer, Chelate vs. Non-Chelate	Z,*E* Isomerization *
	ΔG, kcal/mol		
Li^+^	−15.00	−3.22	6.93
Na^+^	−12.12	−5.36	1.11

* The ΔG values for *Z*,*E* isomerization were calculated from the free energies of chelated forms.

## Data Availability

The data are contained within the article.
